# Response to ‘Comment on “Quantum correlations are weaved by the spinors of the Euclidean primitives”’

**DOI:** 10.1098/rsos.220147

**Published:** 2022-11-16

**Authors:** Joy Christian

**Affiliations:** Einstein Centre for Local-Realistic Physics, Oxford, UK

**Keywords:** quantum correlations, local causality, Bell’s theorem, spinors, quaternions, normed division algebras

## Abstract

In this paper, I respond to a critique of one of my papers previously published in the *Royal Society Open Science* entitled ‘Quantum correlations are weaved by the spinors of the Euclidean primitives.’ Without engaging with the geometrical framework presented in my paper, the critique incorrectly claims that there are mathematical errors in it. I demonstrate that the critique is based on a series of misunderstandings, and refute each of its claims of error. I also bring out a number of logical, mathematical and conceptual errors from the critique and the critiques it relies on.

## 1. Introduction

The geometric framework proposed in my paper [1] is based on a Clifford-algebraic interplay between a quaternionic 3-sphere, or *S*^3^, which I have taken to model the geometry of three-dimensional physical space, and an octonion-like 7-sphere, or *S*^7^, which is an algebraic representation space of this quaternionic 3-sphere. This framework overcomes Bell’s theorem by reproducing quantum correlations local-realistically as geometrical effects, without resorting to backward causation, superdeterminism or any conspiracy loophole. It is summed up in the following theorem proven in [1]:

**Theorem 3.1.**

*Every quantum mechanical correlation can be understood as a classical, local, realistic and deterministic correlation among a set of points of an octonion-like 7-sphere.* ▪

It is important to note that, contrary to the impression given in the critique [2], it has not engaged with the actual contents of the framework presented in [1]. The readers are urged to read [1] for themselves to appreciate this fact. For a comprehensive review and detailed mathematical foundations of the 7-sphere framework for reproducing quantum correlations as geometrical effects, see also [3].

It is also important to note that I have already refuted many incorrect claims made by the same author of the critique [2] in several of my previous publications [4–7]. But since these claims have been repeated in [2], some overlap in my point-by-point response to them below is not avoidable.

## 2. Point-by-point response to the critique [2]

### 2.1. Incorrect claims in the first paragraph of the critique

Let me begin with a number of incorrect claims and mistakes in the first paragraph of the critique.

(1) It is claimed in the first paragraph of [2] that in my papers published between 2007 and 2021, I have proposed ‘a *local hidden variable model* (but not always the same one) … ’ However, in all my publications on the subject [5–10], what I have proposed is one and the same quaternionic 3-sphere model mentioned above. The critique’s claim is thus an early indication that it has not quite understood what I have proposed in my papers [5–10].
(2) This fact is confirmed as we read the paragraph further, which claims: ‘Christian argues that Bell’s proof of his theorem is mathematically wrong.’ But nowhere have I made such a claim. The mathematical inequalities on which Bell’s argument depends are trivially correct (cf. eqn (4.9) of [1]). In fact, they were discovered and proven by Boole some 111 years before the work of Bell [11,12]. I have never questioned Boole’s inequalities in any of my publications. Thus the claim made in the critique is not correct.
(3) The next mistake in the Introduction is more serious. It is claimed that in my paper [1] I connect the 3-sphere, or *S*^3^, ‘to special relativity, specifically to the solution of Einstein’s field equations known as Friedmann–Robertson–Walker space–time with a constant spatial curvature.’ The same claim appears in several preprint versions of the critique [2] that have been posted on arXiv as well as in online discussions. The quoted sentence from [2] thus exhibits a lack of understanding of the difference between the special and general theories of relativity and how my proposed quaternionic 3-sphere model fits into the Friedmann–Robertson–Walker solution of Einstein’s field equations of *general* relativity. In fact, much of the confusion in [2] stems from its failure to understand the difference between strong correlations within flat space–time IR × IR^3^ and curved space–time IR × *S*^3^.
(4) There is a further mathematical error in the first paragraph of [2]. Its last sentence reads: ‘He furthermore connects it to the 7-sphere *S*^7^, thought of as a quaternionic 3-sphere rather than a real 3-sphere.’ The two parts of the quoted sentence are even dimensionally incorrect. A 7-sphere cannot be thought of as 3-sphere in any sense. Their dimensions are not the same. Such misreadings of elementary facts indicate that critique’s reading of [1] is mistaken and what it claims to be errors in [1] are, in fact, errors in the reading of [1].

### 2.2. Less than correct narrative and claims in the rest of its Introduction

In the rest of its Introduction, the critique [2] makes several claims of equally questionable merit:

(1) It claims that in [13], a critique of my work was published. However, what is published in [13] is not a critique of my work at all. In it, a different model is presented based on a flat space, and using matrices and vector algebra, instead of the quaternionic 3-sphere model using Geometric Algebra I have proposed in my work [5–10]. This alternative model is then criticized, claiming the criticism to be that of my model. The critique in [13] is thus oblivious to the difference between its own flat space model and my 3-sphere model, and to the fact that such a comparison amounts to committing a logical fallacy. Moreover, in the abstract of [13], it is claimed that ‘Christian’s fundamental idea is simple and quite original: he gives a probabilistic interpretation of the fundamental GA equation *a* · *b* = (*ab* + *ba*)/2.’ However, I have not proposed any such interpretation anywhere in my writings. Unfortunately, this is not the only thing that is mistaken in [13]. Several other equations attributed to my work are also misconstrued in [13]. For example, nowhere in my writings does there appear anything like eqn (22) stipulated in [13]. It also does not follow mathematically from any other equations I have written down anywhere, without violating the conservation of the initial zero spin angular momentum, as I have explained, for example, in Subsection IV E of [6], Subsection III E of [7] and Section VIII of [9]. There are also other oversights in [13], which I have brought out in [4–7]. In particular, [13] makes the same mathematical mistakes I bring out in detail below in §2.7.
(2) In the Introduction of [2], it is claimed that other papers have also refuted my work. But to date, no one has refuted any part of my work, or undermined it in any way. To be sure, there have been attempts of refutation, but I have elucidated the errors in all such claims, for example, in [4–7] and references cited therein. See, especially, ch. 9 to 12 in [5].
(3) It is further claimed in the Introduction of [2] that Lasenby in [14] has independently made the same claims about the algebraic core of the 7-sphere framework proposed in [1]. However, in [14], it is acknowledged that ‘ … several of the points made [in [14]] have been made independently by [the author of [2]] and others in the discussion thread attached to the Royal Society paper … ’ Thus it is not surprising that the claims in [14] are similar to those in [2]. More importantly, in [3] and [15], I have refuted all of the claims made in [14].
(4) The Introduction in [2] is summed up by quoting a few disjoint sentences out of context from Section II of my reply in [6]. This allows [2] to misrepresent the argument I have presented in Section II of [6] concerning Bell’s so-called ‘theorem’, to which I now turn.

### 2.3. Bell’s ‘theorem’ is not a theorem in the mathematical sense

One of the most serious misconceptions exhibited in the critique [2] is its presumption that Bell’s so-called ‘theorem’ is a proven theorem in the mathematical sense and therefore any critique of it must be wrong. But it is important to appreciate the difference between the mathematical inequalities used by Bell and his physical argument based on those inequalities. As noted above, the mathematical inequalities discovered by Boole [11,12] on which Bell’s theorem depends are trivially correct. Moreover, while even proven mathematical theorems may not be immune to refutations, as so lucidly explained by Lakatos [16], Bell’s theorem is not a theorem in the mathematical sense to begin with. It depends on a number of implicit and explicit *physical* assumptions, which can be and have been questioned before, not only by me [1,5–10], but also by many others (cf. footnote 1 in [9]). If it were a theorem in the mathematical sense, then it would not require physical experiments for its validity and any loophole (or ‘gap’) would render it invalid.

It is important to note that Bell’s own writings do not exhibit any such misconception. Indeed, he actively sought strategies to overcome his ‘theorem'. In the concluding sentence of ch. 17 of his book [17], Bell reminds us that ‘ … what is proved by impossibility proofs is lack of imagination.’ More seriously, in §8 of ch. 7 and §10 of ch. 24 of his book, Bell points out that his theorem depends on the assumption of experimenters’ ‘free will’, which may turn out to be illusory. Thus Bell was well aware of the other physical and metaphysical assumptions that are necessary to support his theorem, in addition to those of locality and realism.

In the Introduction and §4.2 of [1], and in Section II of [6] and Section III of [7], I have highlighted several other physical assumptions that are necessary for Bell’s theorem to hold. Among these, there are two assumptions that have been hitherto underappreciated, even though Bell himself has discussed them at least indirectly. Let me bring them out in some detail for clarity.

### 2.4. Assumption of the additivity of expectation values

The first among the two assumptions is the assumption of the additivity of expectation values:

2.1 $$\begin{array}{rl} & \int_{\Lambda }A(a,\;\lambda )B(b,\;\lambda )\;p(\lambda )\;d\lambda +\int_{\Lambda }A(a,\;\lambda )B(b^{\prime },\;\lambda )\;p(\lambda )\;d\lambda \\ & \;+\int_{\Lambda }A(a^{\prime },\;\lambda )B(b,\;\lambda )\;p(\lambda )\;d\lambda -\int_{\Lambda }A(a^{\prime },\;\lambda )B(b^{\prime },\;\lambda )\;p(\lambda )\;d\lambda \\ & =\int_{\Lambda }\{\;A(a,\;\lambda )\;B(b,\;\lambda )+A(a,\;\lambda )\;B(b^{\prime },\;\lambda )+A(a^{\prime },\;\lambda )\;B(b,\;\lambda )-A(a^{\prime },\;\lambda )\;B(b^{\prime },\;\lambda )\;\}\;p(\lambda )\;d\lambda , \end{array}$$

where Λ is the space of hidden variables *λ*, and *p*(*λ*) is the probability distribution of *λ*, so that

2.2 $$E(a,b)=\int_{\Lambda }A(a,\;\lambda )\;B(b,\;\lambda )\;p(\lambda )\;d\lambda ,$$

which is the usual expression of expectation value in the context of Bell’s theorem [17]. Now it is universally accepted that without the validity of equation (2.1), the Bell-CHSH inequalities cannot be derived. On the other hand, given the assumption of experimenters’ ability to freely, randomly, or spontaneously choose the detector directions **a** and **b**, which amounts to assuming *p*(*λ* | **a**, **b**) = *p*(*λ*), mathematically equation (2.1) follows at once. However, physically equation (2.1) harbours a non-trivial assumption. This is obliquely recognized in the critique [2] in the last paragraph of its §2.2:

He also argues [in [18]] that Bell’s proof contains a fundamental error in reasoning: the Bell-CHSH inequality involves correlations obtained from different sub-experiments involving measurements of non-commuting observables, and (he says) therefore cannot be combined. However, in quantum mechanics, even if two observables do not commute, a real linear combination of those observables is another observable. By the linearity encapsulated in the basic rules of quantum mechanics, expectation values of linear combinations of non-commuting observables are the same linear combination of the expectation values of each observable separately. If a local hidden variables model reproduces the statistical predictions of quantum mechanics, then it must reproduce this linearity.

But the last sentence of the quoted paragraph is manifestly incorrect. It reveals a profound lack of understanding of what is meant by a hidden variable theory since the pioneering work of von Neumann [19]. I have discussed the problem with equation (2.1) in Section II of [6] and in [18]. While mathematically correct, equation (2.1) is physically meaningless within any hidden variable theory. It is an assumption over and above those of locality and realism. In fact, it is the same physical mistake that von Neumann’s former theorem against general hidden variable theories harboured. For observables that are not simultaneously measurable, such as those involved in Bell-test experiments, the replacement of the sum of expectation values with the expectation value of the sum, although respected in quantum mechanics, does not hold for hidden variable theories. This was pointed out by Einstein and Grete Hermann in the 1930s within the context of von Neumann’s theorem, and 30 years later by Bell [20] and others, as I have explained in [18].

The example Bell [20] gives to illustrate this problem is that of the spin components of a fermion. A measurement of *σ*_*x*_ can be made with a suitably oriented Stern–Gerlach magnet. But the measurement of *σ*_*y*_ would require a different orientation of the magnet. And the measurement of the sum (*σ*_*x*_ + *σ*_*y*_) would again require a third and quite a different orientation of the magnet from the previous two. Consequently, the result of the last measurement—i.e. an eigenvalue of (*σ*_*x*_ + *σ*_*y*_)—will not be the sum of an eigenvalue of *σ*_*x*_ plus that of *σ*_*y*_. The additivity of the expectation values, namely, 〈*ψ* | *σ*_*x*_ | *ψ*〉 + 〈*ψ* | *σ*_*y*_ | *ψ*〉 = 〈*ψ* | *σ*_*x*_ + *σ*_*y*_ | *ψ*〉, is a peculiar property of the quantum states |*ψ*〉. It would not hold for individual eigenvalues of non-commuting observables in a dispersion-free state of a hidden variable theory. In a dispersion-free state, every observable would have a unique value equal to one of its eigenvalues. And since there can be no linear relationship between the eigenvalues of non-commuting observables, the additivity relation that holds for quantum mechanical states would not hold for dispersion-free states [18].

In summary, the problem with equation (2.1) is that, while the sum of expectation values appearing on its left-hand side is mathematically equal to the expectation value of the sum appearing on its right-hand side, and while this equality holds in quantum mechanics, it does not hold for hidden variable theories based on dispersion-free states [20]. That is because the eigenvalue of a sum of operators is not the sum of eigenvalues when the constituent operators are non-commuting, as in Bell-test experiments. In other words, while the joint results A(a,λ)B(b,λ), etc. on the left-hand side of equation (2.1) are possible eigenvalues of the spin operators ***σ***_1_ · **a** ⊗ ***σ***_2_ · **b**, etc., their summation

2.3 $$A(a,\;\lambda )\;B(b,\;\lambda )+A(a,\;\lambda )\;B(b^{\prime },\;\lambda )+A(a^{\prime },\;\lambda )\;B(b,\;\lambda )-A(a^{\prime },\;\lambda )\;B(b^{\prime },\;\lambda )$$

appearing as the integrand on the right-hand side of equation (2.1) is *not* an eigenvalue of the operator

2.4 $$\sigma_{1}\cdot a\;⊗\;\sigma_{2}\cdot b+\sigma_{1}\cdot a\;⊗\;\sigma_{2}\cdot b^{\prime }+\sigma_{1}\cdot a^{\prime }\;⊗\;\sigma_{2}\cdot b-\sigma_{1}\cdot a^{\prime }\;⊗\;\sigma_{2}\cdot b^{\prime },$$

because the joint operators ***σ***_1_ · **a** ⊗ ***σ***_2_ · **b**, etc. do not commute with each other. On the other hand, the very meaning of a hidden variable theory dictates simultaneous assignment of definite eigenvalues to *all* observables of the singlet system [18], *including* the one in (2.4), whether or not they are actually measured (albeit this assignment has to be contextual in the light of the Kochen–Specker theorem). But since the sum of results (2.3) is not one of the eigenvalues of the summed operator in (2.4), its appearance on the right-hand side of (2.1) is incorrect, making the replacement of the left-hand side of equation (2.1) with its right-hand side at least physically invalid. But without this replacement the absolute upper bound of 2 on the left-hand side of equation (2.1) cannot be derived.

Once this oversight is removed from Bell’s ‘theorem’ [17] and local realism is implemented correctly by using the correct eigenvalue of (2.4) (which I have worked out explicitly in appendix A of [18]) instead of (2.3) on the right-hand side of (2.1), the bounds on the left-hand side of (2.1) work out to be $\pm 2\sqrt{2}$ instead of ±2 (as I have demonstrated, for example, in Section V of [18]), thereby mitigating the conclusions of Bell’s theorem [18]. Consequently, what is ruled out by the Bell-test experiments is not local realism as widely believed, but the assumption of the additivity of expectation values, which does not hold in general for any hidden variable theories to begin with.

### 2.5. Assumption of a flat and immutable space–time

The second assumption necessary to support Bell’s theorem is that of immutable space–time. The formulation of the theorem thus neglects the mutable space–time geometries of Einstein’s theory of gravity. Note that it is not the strength of gravity that is at stake here but the qualitative differences between immutable flat space–time and mutable curved space–times [21]. This is hinted at by Bell himself. In §8 of ch. 7 of his book [17], while exploring possible strategies that may be used to overcome his theorem, he writes: ‘The space–time structure has been taken as given here. How then about gravitation?’ Thus Bell seems to have anticipated using a solution of Einstein’s field equations of general relativity to overcome his theorem, as I have proposed in [1,5–10,22].

By contrast, in the critique [2], Bell’s theorem is stated using ‘ordinary three-dimensional Euclidean space’:

Suppose that *X*_*a*_ and *Y*_*b*_ are a family of random variables on a single probability space, taking values in the set {−1, +1}, and where *a* and *b* denote directions in ordinary three-dimensional Euclidean space, represented by unit vectors *a*, *b*. Then it is not possible that $E(X_{a}Y_{b})=-a\cdot b$ for all *a* and *b*.

Stated thus, the physical limitation of Bell’s theorem is conspicuous. It is revealed in the following explicit assumption about the geometry of the three-dimensional physical space: ‘ … where *a* and *b* denote directions in ordinary three-dimensional Euclidean space, represented by unit vectors *a*, *b*.’ But why must we allow such an outmoded view of physical space to make radical claims about the fundamental nature of locality and reality after more than a century of general-relativistic revolution in which space–time geometry is rendered dynamical and malleable? And why must we use vector ‘algebra’ after more than a century and a half of insights from Grassmann and Clifford on the correct algebraic representation of the three-dimensional physical space? As I have proposed in [1,5–10], there are both theoretical and observational reasons that compel us to model physical space as a closed and compact quaternionic 3-sphere, or *S*^3^, instead of a flat Euclidean space, or IR^3^, both being admissible spatial parts of one of the well-known cosmological solutions of Einstein’s field equations of general relativity. Moreover, as explained in [1,5–10], the correct language to model *S*^3^ as physical space is Geometric Algebra, not vector ‘algebra'. But once the physical space is modelled as *S*^3^ instead of IR^3^ using the powerful language of Geometric Algebra, the correlation between the local results $A_{a}$ and $B_{b}$ observed by Alice and Bob inevitably turns out to be $E(A_{a}B_{b})=-a\cdot b$ as I have proved in [1,5–10,22], contrary to the claims made in [2]. For a comprehensive proof of $E(A_{a}B_{b})=-a\cdot b$ within the local-realistic geometry of *S*^3^, I especially recommend the derivations of the singlet correlations in [3,22].

Given the many implicit assumptions required to prove Bell’s theorem, in addition to locality and realism, I have brought out in the Introduction and §4.2 of [1], in Section II of [6] and in Section III of [7], together with the two assumptions discussed above, the infallibilist view of the theorem adhered to in the critique [2] (to borrow the term coined by Lakatos [16]) is not justified.

### 2.6. Orientation *λ* of $K^{\lambda }$ acts as a hidden variable in the *S*^7^ model

Another issue raised in the critique [2] concerns the orientation of the vector space $K^{\lambda }$ used in [1]:

A curious elementary mathematical error is that he defines two algebras, built from two eight-dimensional real vector spaces $K^{+}$ and $K^{-}$ by specifying a vector space basis for each algebra and multiplication tables for the eight basis elements of each algebra. But they are the *same* algebra. The linear spans of those two bases are trivially the same. The multiplication operation is the same.

However, there is no such error in [1]. I have not defined ‘two algebras’ built from ‘two’ vector spaces $K^{+}$ and $K^{-}$ and multiplication tables for ‘each algebra'. I have defined only *one* algebra, namely the even sub-algebra $K^{\lambda }$ of the Clifford algebra Cl_4,0_, with only *one* multiplication table for that algebra, namely table 1 on p. 8 of [1]. The superscript *λ* = ± over K refers to the orientation (or handedness) of the corresponding vector space. Moreover, there is no claim in [1] that the linear spans of $K^{+}$ and $K^{-}$ are different. $K^{\lambda }$ is one and the same algebra, or vector space, with two possible orientations. There is nothing unusual or unorthodox about this concept. It is clearly explained in §2.3 of [1] that $K^{+}$ and $K^{-}$ differ only in their orientations: *λ* = + or *λ* = −. If one of them (say $K^{+}$) is deemed right-handed, then the other one ($K^{-}$) is left-handed, and vice versa, with the definition of orientation from Milnor [23] reproduced on p. 7 of [1]:

*Definition of Orientation*: An orientation of a finite-dimensional vector space $V_{n}$ is an equivalence class of ordered basis, say {*b*_1_, …, *b*_*n*_}, which determines the same orientation of $\;V_{n}$ as the basis {*b*′_1_, …, *b*′_*n*_} if *b*′_*i*_ = *ω*_*ij*_ *b*_*j*_ holds with det(*ω*_*ij*_) > 0, and the opposite orientation of $V_{n}$ as the basis {*b*′_1_, …, *b*′_*n*_} if *b*′_*i*_ = *ω*_*ij*_ *b*_*j*_ holds with det(*ω*_*ij*_) < 0.

On the p. 8 of [1], I have stated: ‘It is easy to verify that the bases of $K^{+}$ and $K^{-}$ are indeed related by an 8 × 8 diagonal matrix whose determinant is (−1)^7^ < 0. Consequently, $K^{+}$ and $K^{-}$ indeed represent right-oriented and left-oriented vector spaces, respectively, in accordance with our definition of orientation. We can therefore leave the orientation unspecified and write $K^{\pm }$ as $K^{\lambda }=\;\mathrm{span}\{1,\;\lambda e_{x}e_{y},\;\lambda e_{z}e_{x},\;\lambda e_{y}e_{z},\;\lambda e_{x}e_{\infty },\;\lambda e_{y}e_{\infty },\;\lambda e_{z}e_{\infty },\;\lambda I_{3}e_{\infty }\},\;\lambda^{2}=1⟺\lambda =\pm 1.$’

In the later sections in [1], *λ* is taken as a Bell-type hidden variable, with physical consequences. Therefore, $K^{+}$ and $K^{-}$ are physically not identical within the 7-sphere framework proposed in [1].

### 2.7. Algebra $K^{\lambda }$ used in [1] is not incompatible with Hurwitz’s theorem

In its §2, the critique [2] claims that Hurwitz’s theorem contradicts the mathematical claims in [1]. However, the critique seems to have missed appendix A of [1] where I have explicitly discussed Hurwitz’s theorem in detail, as well as its significance for the 7-sphere framework presented in [1]. Overlooking this discussion, the critique claims a counterexample to eqn (2.40) of [1], which says that for any multivectors *X* and *Y* in $K^{\lambda }$, the following composition law holds:

2.5 ‖XY‖=‖X‖ ‖Y‖,

where the norms defined in [1] as $\|X\|\;:=\sqrt{XX^{†}\;}$ using geometric products are positive definite:

2.6 ‖X‖=0 ⟺ X=0.

This definition is consistent with how norms are defined for complex numbers *c*, quaternions **q** and octonions **O**; namely, by $\|c\|=\sqrt{c\;c^{†}}$, $\|q\|=\sqrt{qq^{†}}$, and $\|O\|=\sqrt{OO^{†}}$, respectively [1,24]. Moreover, a geometric product between *X* and $X^{†}$ in the definition $\|X\|\;:=\sqrt{XX^{†}\;}$ is necessary for maintaining consistency between the two sides of equation (2.5) because only a geometric product between *X* and *Y* can produce a new multivector *Z* = *XY* in $K^{\lambda }$ appearing on its left-hand side. On the other hand, because the algebra $K^{\lambda }$ is a tensor product $H⊗C^{\prime }$ of quaternions with split complex numbers, its elements are of the form $X=\;q_{r}+q_{d}\;\varepsilon$ with $\varepsilon^{2}=1$, and consequently the quadratic form $XX^{†}$ in general takes values in split complex numbers $C^{\prime }$ instead of real numbers.

Challenging the norm relation (2.5) (which I have proved in §2.5 of [1], appendix A.1 of [3] and appendix B of [25]), the critique in [2] alleges the following counterexample without providing a definition of norm or specifying what it means by ‘taking norms’ mathematically:

However, the author’s algebra has an element called the ‘pseudo-scalar’, I will denote it by *M*, such that *M*^2^ = 1. It follows that 0 = *M*^2^ − 1 = (*M* − 1)(*M* + 1). Taking norms, 0 = ‖*M* − 1‖ . ‖*M* + 1‖. Hence ‖*M* − 1‖ = 0 or ‖*M* + 1‖ = 0. Therefore *M* − 1 = 0 or *M* + 1 = 0, which implies that *M* = 1 or *M* = −1. That is a contradiction.

The same claim appears in [13,26], after the correction of a mistake I had pointed out in [4].

But it is easy to see that this alleged counterexample continues to harbour several mistakes. The first mistake in the quoted claim is immediately obvious. It starts out with the equation *M*^2^ = 1 and ends with the equations *M* = 1 or *M* = −1. And then it claims that ‘That is a contradiction.’ However, the argument $M^{2}=1\Rightarrow M=1$ or *M* = −1 by itself is not a contradiction. To allege a contradiction, one must assume that *M* is a pseudoscalar. But then it by no means follows from ‖*M* − 1‖ ‖*M* + 1‖ = 0 that either ‖*M* − 1‖ = 0 or ‖*M* + 1‖ = 0 as alleged in the critique, unless two different product rules are employed on the two sides of equation (2.5)—a geometric product, namely (*M* − 1)(*M* + 1) = *M*^2^ − 1, to derive 0 on the left-hand side of (2.5) and scalar products such as in $\sqrt{(M-1)\cdot {\left(M-1\right)}^{†}}$ to evaluate the norms on its right-hand side. Needless to say, one can always derive a contradiction from *any* mathematical equation by employing two different product rules on the two sides of that equation.^1^ Barring that inconsistency, what the equation ‖*M* − 1‖ ‖*M* + 1‖ = 0 says is that the *geometric* product between ‖*M* − 1‖ and ‖*M* + 1‖ must vanish, precisely because *M*, and therefore ‖*M* − 1‖ and ‖*M* + 1‖, are no longer scalar quantities, and a geometric product, namely (*M* − 1)(*M* + 1), is used to derive 0 on the left-hand side of (2.5).

Indeed, the notation used in the critique for its equation ‘0 = ‖*M* − 1‖ · ‖*M* + 1‖’ seems to tacitly acknowledge that ‖*M* − 1‖ and ‖*M* + 1‖ are not scalars. For otherwise there would be no need to introduce a ‘dot’ between ‖*M* − 1‖ and ‖*M* + 1‖ indicating a scalar product between them when no such dot appears in the relation (2.5). But even with the scalar product introduced between ‖*M* − 1‖ and ‖*M* + 1‖ in an ad hoc manner, the critique’s alleged conclusion that either ‖*M* − 1‖ = 0 or ‖*M* + 1‖ = 0 does not follow. Therefore, the critique’s claim of contradiction fails.

Now it is easy to prove that geometric product between ‖*M* − 1‖ and ‖*M* + 1‖ does vanish. In [1], I have denoted the pseudoscalar in $K^{\lambda }$ by $\varepsilon \;:=e_{1}e_{2}e_{3}e_{\infty }\neq \pm 1$. It satisfies the properties $\varepsilon^{†}=\varepsilon$ and $\varepsilon^{2}=1$. Given this, what the critique has considered are the following elements in $K^{\lambda }$:

2.7 X=ε−1andY=ε+1.

But, to begin with, no such two-dimensional multivectors play any role whatsoever in the 7-sphere framework presented in [1]. Therefore, even if such ad hoc two-dimensional objects lead to ‘contradiction’ as alleged in the critique [2], that would have no effect on or consequences for the 7-sphere framework. Nevertheless, it is instructive to play along with (2.7). The question then is: do such multivectors in $K^{\lambda }$ satisfy the norm relation (2.5)? If they do not, then the claim made in the critique would be correct. But if they do, then the claim made in my paper would be correct. To investigate the question, we begin with evaluating the left-hand side of the norm relation (2.5):

2.8 $$\|XY\|=\|(\varepsilon -1)(\varepsilon +1)\|=||\varepsilon^{2}-1||=||1-1||=\|\;0\;\|=0,$$

where $\varepsilon^{2}=1$ is used. Next, using $\varepsilon^{†}=\varepsilon$, we evaluate the right-hand side of the norm relation (2.5):

2.9 $$\|X\|=\|(\varepsilon -1)\|=\sqrt{(\varepsilon -1){\left(\varepsilon -1\right)}^{†}}=\sqrt{\left(\varepsilon -1)(\varepsilon -1\right)}=\sqrt{2\;(1-\varepsilon )}=1-\varepsilon$$

and

2.10 $$\|Y\|=\|(\varepsilon +1)\|=\sqrt{(\varepsilon +1){\left(\varepsilon +1\right)}^{†}}=\sqrt{\left(\varepsilon +1)(\varepsilon +1\right)}=\sqrt{2\;(1+\varepsilon )}=1+\varepsilon ,$$

and therefore

2.11 $$\|X\|\;\|Y\|=(1-\varepsilon )(1+\varepsilon )=1-\varepsilon^{2}=1-1=0.$$

Comparing (2.8) and (2.11), we see that the norm relation (2.5) is satisfied for the multivectors *X* and *Y* considered in (2.7). Thus, contrary to the claim in [2], *X* and *Y* considered in (2.7) do not entail a counterexample to (2.5). The contradiction to (2.5) is achieved in [2] by computing norms incorrectly. Moreover, the non-scalar values of ‖*X*‖ and ‖*Y*‖ arrived at in (2.9) and (2.10) reiterate the fact that *X* and *Y* considered in the critique [2] are not parts of the 7-sphere framework proposed in [1].

### 2.8. Proof of the norm relation (2.5) for the eight-dimensional algebra $K^{\lambda }$

Elsewhere [3,25], I have proved that the norm relation (2.5) holds, without exception, for arbitrary *X* and *Y* in $K^{\lambda }$, and, as a special case, reduces to the one with scalar values for ‖*X*‖, ‖*Y*‖ and ‖*XY*‖. The proofs of (2.5) and (2.6) are straightforward.^2^ As noted above, the algebra $K^{\lambda }$ is a tensor product $H⊗C^{\prime }$ of quaternions with split complex numbers [24], but with conjugation (or ‘reverse’) affecting only the quaternions [1]. In other words, any multivectors *X* and *Y* in $K^{\lambda }$ are of the form $X=\;{q\;}_{r1}+{q\;}_{d1}\;\varepsilon$ and $Y=\;{q\;}_{r2}+{q\;}_{d2}\;\varepsilon$ with $\varepsilon^{2}=+1$ and $\varepsilon^{†}=\varepsilon$, where **q **_*r*1_ and **q **_*d*1_ constituting *X*, for example, are two independent quaternions, which can be written as **q **_*r*1_ = *g*_1_ + *I*_3_**u**_1_ and **q **_*d*1_ = *h*_1_ + *I*_3_**v**_1_, where *g*_1_ and *h*_1_ are scalars, *I*_3_ = **e**_1_**e**_2_**e**_3_ is the standard pseudoscalar in three dimensions, and **u**_1_ = *u*_1*x*_**e **_*x*_ + *u*_1*y*_**e **_*y*_ + *u*_1*z*_**e **_*z*_ and **v**_1_ = *v*_1*x*_**e **_*x*_ + *v*_1*y*_**e **_*y*_ + *v*_1*z*_**e **_*z*_ are Cartesian vectors. As a result, the geometric product $XX^{†}$ between *X* and $X^{†}$ works out to be

2.12 $$XX^{†}=({q\;}_{r1}+{q\;}_{d1}\;\varepsilon ){\left({q\;}_{r1}+{q\;}_{d1}\;\varepsilon \right)}^{†}$$

2.13 $$=({q\;}_{r1}\;q_{r1}^{†}+{q\;}_{d1}\;q_{d1}^{†})+({q\;}_{r1}\;q_{d1}^{†}+{q\;}_{d1}\;q_{r1}^{†})\;\varepsilon$$

2.14 $$=(g_{1}^{2}+u_{1}\cdot u_{1}+h_{1}^{2}+v_{1}\cdot v_{1})+(2\;g_{1}h_{1}+2\;u_{1}\cdot v_{1})\;\varepsilon$$

2.15 =(a scalar) +(a scalar) ε.

And likewise, using $Y=\;{q\;}_{r2}+{q\;}_{d2}\;\varepsilon$, together with **q **_*r*2_ = *g*_2_ + *I*_3_**u**_2_ and **q **_*d*2_ = *h*_2_ + *I*_3_**v**_2_, the geometric product $YY^{†}$ between *Y* and $Y^{†}$ works out to be

2.16 $$YY^{†}=({q\;}_{r2}+{q\;}_{d2}\;\varepsilon ){\left({q\;}_{r2}+{q\;}_{d2}\;\varepsilon \right)}^{†}$$

2.17 $$=({q\;}_{r2}\;q_{r2}^{†}+{q\;}_{d2}\;q_{d2}^{†})+({q\;}_{r2}\;q_{d2}^{†}+{q\;}_{d2}\;q_{r2}^{†})\;\varepsilon$$

2.18 $$=(g_{2}^{2}+u_{2}\cdot u_{2}+h_{2}^{2}+v_{2}\cdot v_{2})+(2\;g_{2}h_{2}+2\;u_{2}\cdot v_{2})\;\varepsilon$$

2.19 =(a scalar) +( a scalar) ε.

The positive definiteness (2.6) is now easy to prove. Since $X=\;{q\;}_{r1}+{q\;}_{d1}\;\varepsilon$, it can be zero only if both **q **_*r*1_ = 0 and **q **_*d*1_ = 0. But from (2.13) that implies $XX^{†}=0$, and therefore $\sqrt{XX^{†}}=\|X\|=0$. Thus X=0 ⟹‖X‖=0 is proved. Conversely, suppose ‖*X*‖ = 0. Then again from (2.13) we have $\|X\|=\sqrt{XX^{†}}=\sqrt{({q\;}_{r1}\;q_{r1}^{†}+\;{q\;}_{d1}\;q_{d1}^{†})+({q\;}_{r1}\;q_{d1}^{†}+\;{q\;}_{d1}\;q_{r1}^{†})\;\varepsilon \;}=0$. But that is possible only if the quantities appearing in both parentheses under the square root are zero. However, both ${q\;}_{r1}\;q_{r1}^{†}$ and ${q\;}_{d1}\;q_{d1}^{†}$ in the first parenthesis $\left({q\;}_{r1}\;q_{r1}^{†}+\;{q\;}_{d1}\;q_{d1}^{†}\right)$ are positive definite scalars, and therefore they both must be zero for ${q\;}_{r1}\;q_{r1}^{†}+\;{q\;}_{d1}\;q_{d1}^{†}$ to be zero. But since ${q\;}_{r1}\;q_{r1}^{†}$ and ${q\;}_{d1}\;q_{d1}^{†}$ are positive definite scalars, they can be zero only if **q **_*r*1_ and **q **_*d*1_ are zero. In other words, both **q **_*r*1_ and **q **_*d*1_ must be zero for ‖*X*‖ = 0 to hold. But **q **_*r*1_ = 0 and **q **_*d*1_ = 0 implies that $X=\;{q\;}_{r1}+{q\;}_{d1}\;\varepsilon =0$. Therefore, the converse ‖X‖=0 ⟹X=0 holds. The positive definiteness (2.6) is thus proved.

The proof of the norm relation (2.5) is also straightforward. Evidently, the geometric products $XX^{†}$ and $YY^{†}$ written above resemble split complex numbers, because for any two quaternions (such as **q **_*r*1_ and **q **_*d*1_) the quantities appearing in the parentheses in (2.14) and (2.18) are scalar quantities. Thus the products are of the form $XX^{†}=a+b\varepsilon$ and $YY^{†}=c+d\varepsilon$, where $a={q\;}_{r1}\;q_{r1}^{†}+{q\;}_{d1}\;q_{d1}^{†}$, $b={q\;}_{r1}\;q_{d1}^{†}+{q\;}_{d1}\;q_{r1}^{†}$, $c={q\;}_{r2}\;q_{r2}^{†}+{q\;}_{d2}\;q_{d2}^{†}$ and $d={q\;}_{r2}\;q_{d2}^{†}+{q\;}_{d2}\;q_{r2}^{†}$ are scalar quantities, with $\varepsilon^{†}=+\varepsilon$ instead of −ε. Consequently, for the left-hand side of (2.5), we have

2.20 $$\|XY\|=\sqrt{(XY){\left(XY\right)}^{†}}=\sqrt{\left(XY)(Y^{†}X^{†}\right)}=\sqrt{X(YY^{†})X^{†}}=\sqrt{X(c+d\varepsilon )X^{†}}$$

2.21 $$=\sqrt{\left(XX^{†})(c+d\varepsilon \right)}$$

2.22 $$=\sqrt{\left(a+b\varepsilon )(c+d\varepsilon \right)}$$

2.23 $$=\sqrt{(ac+bd)+(ad+bc)\;\varepsilon },$$

because, as noted, the pseudoscalar ε satisfies $\varepsilon^{2}=1$ and commutes with every element of $K^{\lambda }$, and consequently the identity ${\left(XY\right)}^{†}=(Y^{†}X^{†})$ for *X* and *Y* in $K^{\lambda }$ is straightforward to verify. On the other hand, the right-hand side of the equation (2.5) also works out to give the same quantity:

2.24 $$\|X\|\;\|Y\|=\left(\sqrt{XX^{†}}\right)\left(\sqrt{YY^{†}}\right)=\sqrt{\left(a+b\;\varepsilon )(c+d\;\varepsilon \right)}=\sqrt{(ac+bd)+(ad+bc)\;\varepsilon }.$$

Comparing (2.23) and (2.24), we see that when product rules are applied consistently on the two sides of equation (2.5) using geometric products, both of its sides work out to be identical to the square-root of the following quantity, which also resembles a split complex or hyperbolic number,

2.25 $$\begin{array}{rl} \{(\varrho_{r1}^{2} & +\varrho_{d1}^{2})(\varrho_{r2}^{2}+\varrho_{d2}^{2})+({q\;}_{r1}\;q_{d1}^{†}+{q\;}_{d1}\;q_{r1}^{†})({q\;}_{r2}\;q_{d2}^{†}+{q\;}_{d2}\;q_{r2}^{†})\}\\ & +\{(\varrho_{r1}^{2}+\varrho_{d1}^{2})({q\;}_{r2}\;q_{d2}^{†}+{q\;}_{d2}\;q_{r2}^{†})+({q\;}_{r1}\;q_{d1}^{†}+{q\;}_{d1}\;q_{r1}^{†})(\varrho_{r2}^{2}+\varrho_{d2}^{2})\}\;\varepsilon , \end{array}$$

thus proving the norm relation (2.5), where $\varrho_{r1}=\sqrt{{q\;}_{r1}\;q_{r1}^{†}}\;$, etc., and the quantities appearing in the two curly brackets are scalar quantities. Comparing (2.13), (2.17) and (2.25), we thus see that the coefficient algebra underlying $K^{\lambda }$ resembles that of split complex numbers^3^ instead of real numbers, and therefore what is just proved does not contradict Hurwitz’s theorem [1,24], contrary to the claims made in the critiques [2,14]. In fact, the result (2.25) holds for any multivectors *X* and *Y* in $K^{\lambda }$, and therefore the norm relation (2.5) holds for any multivectors *X* and *Y* in the algebra $K^{\lambda }$, because it is possible to work out the square-root of a hyperbolic number such as (2.25), as shown in appendix A.1 of [3]. The normalization or orthogonality condition specified in equation (2.54) of [1]—which amounts to setting the coefficient ${q\;}_{r1}\;q_{d1}^{†}+{q\;}_{d1}\;q_{r1}^{†}=0$ in (2.13) and ${q\;}_{r2}\;q_{d2}^{†}+{q\;}_{d2}\;q_{r2}^{†}=0$ in (2.17) so that the norms ‖*X*‖ and ‖*Y*‖ reduce, respectively, to scalar values $\sqrt{\varrho_{r1}^{2}+\varrho_{d1}^{2}}$ and $\sqrt{\varrho_{r2}^{2}+\varrho_{d2}^{2}}$—then reduces the square-root of the hyperbolic number (2.25) to the scalar quantity

2.26 $$\sqrt{\left(\varrho_{r1}^{2}+\varrho_{d1}^{2})(\varrho_{r2}^{2}+\varrho_{d2}^{2}\right)}.$$

We have thus proved the norm relation (2.5) with scalar values, as originally proved in [1] and [25]:

2.27 $$\|XY\|=\sqrt{(\varrho_{r1}^{2}+\varrho_{d1}^{2})(\varrho_{r2}^{2}+\varrho_{d2}^{2})\;}=\|X\|\;\|Y\|.$$

In particular, since the norm relation (2.5) holds for any multivectors *X* and *Y* in $K^{\lambda }$ and therefore also for those with scalar values for their norms, if *X* and *Y* happen to be unit multivectors so that ‖*X*‖ = 1 and ‖*Y*‖ = 1, then (2.27) necessitates that their product *Z* = *XY* will also be a unit multivector, ‖*Z*‖ = ‖*XY*‖ = ‖*X*‖ ‖*Y*‖ = 1 × 1 = 1, contrary to the claims made in [2,14].

Equation (2.27) also leads us to define a scalar-valued norm for any multivector $Q_{z}={q\;}_{r}+{q\;}_{d}\;\varepsilon$ in $K^{\lambda }$,

2.28 $$\|Q_{z}\|\;:=\sqrt{(Q_{z}Q_{z}^{†}){|}_{\;\varepsilon \cdot (Q_{z}Q_{z}^{†})=0}}\;,$$

which gives the same value for the norm as that obtained using the traditional ad hoc definition in Geometric Algebra (cf. eqn (2.8) in [1]). This is explained also with more detail in [25]. As explained in eqns (2.54)–(2.56) in [1], the quadratic form in the definition (2.28) with split-complex image is

2.29 $$Q_{z}Q_{z}^{†}=({q\;}_{r}\;q_{r}^{†}+{q\;}_{d}\;q_{d}^{†})+({q\;}_{r}\;q_{d}^{†}+{q\;}_{d}\;q_{r}^{†})\varepsilon =(\varrho_{r}^{2}+\varrho_{d}^{2})+({q\;}_{r}\;q_{d}^{†}+{q\;}_{d}\;q_{r}^{†})\varepsilon$$

and $\varepsilon \cdot (Q_{z}Q_{z}^{†})={q\;}_{r}\;q_{d}^{†}+{q\;}_{d}\;q_{r}^{†}$ is the scalar coefficient of ε. It is easy to see that definition (2.28) gives a scalar value for the norm as the Pythagorean distance from the origin in eight dimensions,

2.30 $$\|Q_{z}\|=\sqrt{\varrho_{r}^{2}+\varrho_{d}^{2}}=\sqrt{g^{2}+u_{x}^{2}+u_{y}^{2}+u_{z}^{2}+h^{2}+v_{x}^{2}+v_{y}^{2}+v_{z}^{2}},$$

with the notation **q **_*r*_ = *g* + *I*_3_**u** and **q **_*d*_ = *h* + *I*_3_**v**, and therefore the norms defined by (2.28) are also positive definite: $\|Q_{z}\|=0\;⟺\;Q_{z}=0$. More importantly, definition (2.28) immediately leads to the composition law (2.27) with scalar values. Consequently, the algebra $K^{\lambda }$ considered in [1] is a *normed division algebra* with respect to the norm defined in (2.28), as I have proved in [3] and [25].

Moreover, the definition (2.28) allows us to construct the following 7-sphere embedded in $K^{\lambda }$:

2.31 $$K^{\lambda }↩S^{7}=\left\{Q_{z}=\;q_{r}+q_{d}\;\varepsilon |\|Q_{z}\|=\sqrt{(Q_{z}Q_{z}^{†}){|}_{\;{q\;}_{r}\;q_{d}^{†}+{q\;}_{d}\;q_{r}^{†}=0}}=\sqrt{\varrho_{r}^{2}+\varrho_{d}^{2}}\right\}.$$

Theorem 3.1 stated in the Introduction and proved in [1] refers to this 7-sphere. It is also worth noting that definition (2.28) of the norm is valid also for the real and complex numbers, quaternions and octonions, because for them the coefficient of ε is naturally zero, where the complex numbers and quaternions are isomorphic, respectively, to the even sub-algebras of the Clifford algebras Cl_2,0_ and Cl_3,0_, whereas the algebra $K^{\lambda }$ is isomorphic to the even sub-algebra of the 16-dimensional algebra Cl_4,0_. Thus, the even sub-algebras of the Clifford algebras Cl_1,0_, Cl_2,0_, Cl_3,0_ and Cl_4,0_ form *associative* norm division algebras with respect to the norm defined in (2.28), isomorphic, respectively, to R, C, H and $K^{\lambda }$, in the only possible dimensions 1, 2, 4 and 8 [1,3,25].

### 2.9. The *S*^7^ model concerns the workings of Nature, not computers

The critique [2] puts forward two arguments that it claims are computer science versions of ‘Bell’s core result'. But these arguments are as devoid of physical content as ‘Bell’s core result’, which is simply Boole’s inequalities discussed in §2.3 above. They ignore the obvious fact that Alice and Bob are confined to perform their experiments within a three-dimensional physical space that is common to both of them. If this space is implicitly assumed to be a flat Euclidean space in the arguments put forward in [2], then they are subject to the same criticism I have presented in §2.5. In particular, there is no indication that the geometry and topology of the quaternionic 3-sphere shared between Alice and Bob have been taken into account in either of the arguments. Therefore, they are not relevant for the physical experiments performed by Alice and Bob even within IR^3^. To put this more charitably, at best the arguments in [2] prove the poverty of computer programs that fail to capture the correct geometrical properties of *S*^3^ and *S*^7^, and therefore it is not surprising that they fail to reproduce the observed strong correlations.

By contrast, it is evident from the detailed, line-by-line annotations included in the codes of the two numerical simulations presented in [1] that they are faithful implementations of the physically motivated *S*^7^ model in which *S*^7^ is the algebraic representation space of a quaternionic 3-sphere. In particular, contrary to the claim made in the critique [2], the following line in the code,

if (lambda==1) {q=NA NB;} else {q=NB NA;},

is a correct implementation of the orientation *λ* in the 7-sphere model presented in [1]. It switches the order of the spin bivectors with respect to that of the detector bivectors, thereby shuffling the alternative orientations of the 7-sphere, precisely as necessitated in the model. In other words, the line in question is necessitated by the model itself. In fact, the line in question is *the very essence* of the 7-sphere model. Moreover, by now the codes have been independently translated by several programmers into different computer languages, such as Python, Maple, R and Mathematica [6,7].

It is, however, important to note that the computer codes included in [1] are, by themselves, *not* the model, or even proofs of the model. Their purpose is to demonstrate how the model works. They are pedagogical tools that demonstrate the analytical derivations presented in [1]. Needless to say, the analytical derivations stand on their own and do not require numerical simulations for their validity. On the other hand, the numerical simulations in [1] provide additional support to the analytical derivations, because they are both pedagogically and statistically illuminating.

Ultimately, the merits of the geometrical framework presented in [1] can only be judged through Nature. Therefore, in [8,22], I have proposed an experiment, set in a macroscopic domain, that may be able to falsify the 3-sphere hypothesis underlying the framework presented in [1].

## 3. Conclusion

Without engaging with the actual local-realistic framework for reproducing quantum correlations I have proposed in [1], the critique in [2] has claimed that there are errors in my papers published in [1,5–10]. In this response, I have demonstrated that there are no such errors in [1,5–10]. In fact, all of the claims made in [2] are either mistaken or irrelevant to the 7-sphere framework I have presented in [1]. Moreover, I have brought out a number of logical, mathematical, physical and conceptual mistakes from the critique [2] and other arguments it has relied on (such as those in [14]). As a result, the conclusions drawn in [2] are neither valid nor justified. I urge the readers to also consult my refutations in [6,7] of the similar claims made by the author of [2]. Unfortunately, in the critiques [2] and [14] the main message of [1] has been misunderstood and sidelined. Therefore, in [3,22], I have once again reviewed the mathematical and conceptual bases of the local-realistic framework presented in [1,5–10] to make its message more transparent.

## Data Availability

This article has no additional data.

## Footnotes

1 Some reviewers unjustifiably defended this counterexample during the review process of this paper. In addition to [3], my detailed rebuttal to their defence is available online in the Review History published along with this paper. All variants of the alleged counterexample depend on inconsistent application of product rules on the two sides of equation (2.5). For example, in the counterexample alleged in [14], a contradiction is achieved by employing geometric product between $X=(1+\varepsilon )/\sqrt{2}$ and $Y=(1-\varepsilon )/\sqrt{2}$ on the left-hand side of equation (2.5), whereas scalar products are used to evaluate its right-hand side.

2 During the review process of this paper, one of the reviewers independently verified this proof of the norm relations (2.5) and (2.27) with detailed calculations and comments, which are available online in the Review History published with this paper.

3 Hurwitz’s theorem, as usually stated, assumes that the coefficient algebra underlying the algebras such as R, C, H and O is that of real numbers. It is known, however, that if the norms are generalized to take values in split complex numbers $C^{\prime }=\{a+b\;\varepsilon \;|\;\varepsilon^{2}=1\}$, then new composition algebras can be formed. See the discussion, for example, in §5.3 of [24].
